# TL1A as a Target in Inflammatory Bowel Disease: Exploring Mechanisms and Therapeutic Potential

**DOI:** 10.3390/ijms26115017

**Published:** 2025-05-23

**Authors:** Enrico Tettoni, Roberto Gabbiadini, Arianna Dal Buono, Giuseppe Privitera, Vincenzo Vadalà, Giulia Migliorisi, Peter Bertoli, Alessandro Quadarella, Cristina Bezzio, Alessandro Armuzzi

**Affiliations:** 1 IBD Center, Department of Gastroenterology, IRCCS Humanitas Research Hospital, Via Manzoni 56, 20089 Rozzano, Milan, Italyarianna.dalbuono@humanitas.it (A.D.B.); vincenzo.vadala@humanitas.it (V.V.); giulia.migliorisi@humanitas.it (G.M.);; 2 Department of Gastroenterology and Endoscopy, Fondazione Poliambulanza Istituto Ospedaliero, 25124 Brescia, Brescia, Italy; 3 Department of Biomedical Sciences, Humanitas University, Via Rita Levi Montalcini 4, 20072 Pieve Emanuele, Milan, Italy

**Keywords:** Crohn’s disease, ulcerative colitis, fibrosis, inflammation, new target

## Abstract

Inflammatory bowel diseases (IBD) are chronic disorders characterized by persistent inflammation of the gastrointestinal tract. Despite advances in treatment, a significant proportion of patients remain refractory to current therapies and develop complications, particularly fibrosis, leading to strictures and fistulae. Tumor necrosis factor-like ligand 1A (TL1A) has emerged as a promising new target for IBD treatment, due to its dual role in inflammatory and fibrotic pathways. TL1A, acting through its receptor death receptor 3 (DR3), orchestrates mucosal inflammation by enhancing T-cell activation and promoting pro-inflammatory mediator secretion. TL1A also drives intestinal fibrosis by activating fibroblasts and increasing collagen deposition. Clinical trials evaluating anti-TL1A monoclonal antibodies have shown encouraging efficacy and safety, with significant improvements in clinical remission rates, endoscopic healing, and histologic outcomes. Beyond IBD, TL1A overexpression has been implicated in other immune-mediated inflammatory diseases, highlighting its broader therapeutic potential. This review explores TL1A’s role in IBD pathogenesis, the latest clinical trial data, and its involvement in extraintestinal inflammatory disorders, underscoring its potential as a novel precision-medicine target across multiple diseases.

## 1. Introduction

Inflammatory bowel diseases (IBD), comprising ulcerative colitis (UC) and Crohn’s disease (CD), are chronic and relapsing inflammatory disorders of the gastrointestinal tract that result from a complex interplay of genetic predisposition, immune dysregulation, environmental factors, and gut microbiota alterations [1]. Despite the development of advanced therapies to control intestinal inflammation, a significant proportion of patients remains refractory to current treatments or experiences progressive disease complications, such as strictures and fistulae [2]. These limitations highlight the need for novel therapeutic approaches that can effectively control inflammation and prevent irreversible tissue damage.

Among the emerging therapeutic targets, tumor necrosis factor-like ligand 1A (TL1A), encoded by the *TNFSF15* gene, has garnered considerable attention due to its dual role in inflammatory and fibrotic pathways. TL1A is a member of the tumor necrosis factor (TNF) superfamily and exerts its biological effects through death receptor 3 (DR3, TNFRSF25), which is predominantly expressed on activated lymphocytes and innate immune cells [2,3,4,5,6,7]. TL1A exists in membrane-bound and soluble forms, both of which actively contribute to the regulation of immune responses, T-cell differentiation, and mucosal homeostasis [8,9,10,11]. Genome-wide association studies have identified *TNFSF15* polymorphisms associated with IBD susceptibility and severity, implicating TL1A as a critical orchestrator of the chronic inflammatory state characterizing these diseases [12,13,14,15,16,17,18].

Beyond its involvement in immune activation, TL1A has been implicated in fibrosis, a major complication of CD, where persistent inflammation leads to excessive extracellular matrix deposition and the subsequent formation of intestinal strictures. In vitro models have demonstrated that TL1A signaling promotes fibroblast activation and collagen synthesis [19], contributing to tissue remodeling and fibrotic progression. The ability of TL1A to influence both inflammatory and fibrotic pathways makes it a particularly compelling target for therapeutic intervention in IBD patients.

This review aims to provide a comprehensive assessment of TL1A’s role in IBD pathogenesis, encompassing its molecular pathways, involvement in chronic inflammation and fibrosis, and therapeutic potential. By summarizing preclinical and clinical evidence, we provide an overview of the viability of TL1A blockade as a precision medicine strategy for IBD and related immune-mediated inflammatory conditions.

## 2. TL1A/DR3 Signaling in Mucosal Inflammation

**Figure 1:**
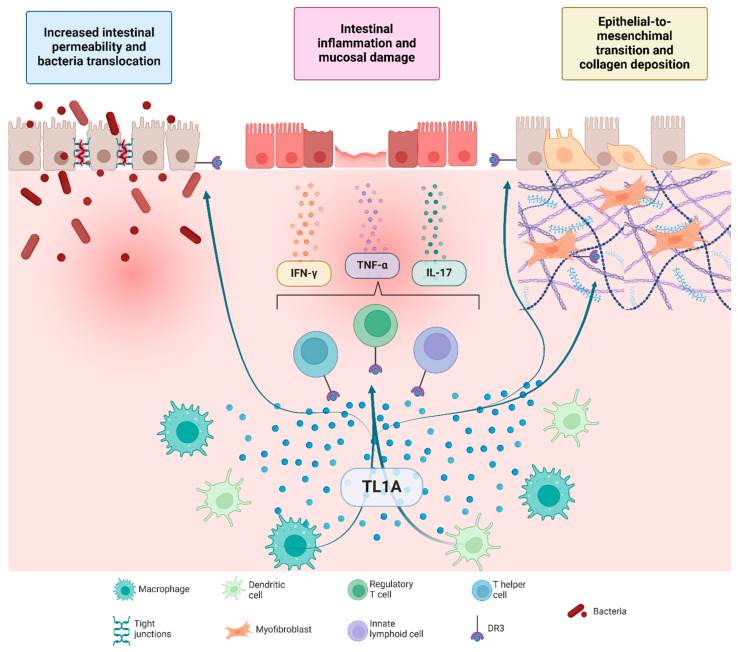
Pathogenetic mechanisms of TL1A in driving intestinal inflammation and fibrosis. TL1A, produced by macrophages and dendritic cells, activates DR3-expressing lymphoid cells, including T helper, regulatory T, and innate lymphoid cells, leading to the release of pro-inflammatory cytokines like IFN-γ, TNF-α, and IL-17. These cytokines drive intestinal inflammation, increase epithelial permeability, and promote bacterial translocation. Chronic activation results in epithelial-to-mesenchymal transition and collagen deposition by myofibroblasts, contributing to fibrosis.

Since its identification in 2002 [20], accumulating evidence has established a substantial link between TL1A and its receptor in driving chronic intestinal inflammation (Figure 1), as commonly observed in patients with IBD. Bamias et al. first showed that both TL1A and DR3 are markedly upregulated at the mRNA and protein levels in inflamed intestinal lesions of CD and UC, as compared to healthy controls, with intermediate signals in unaffected regions [21,22]. These findings have subsequently been confirmed in additional patient cohorts [23,24]. Flow cytometric studies demonstrated that under inflammatory conditions, numerous mucosal cell types—including macrophages, CD4+ T cells, innate lymphoid cells, regulatory T cells, infiltrating plasma cells (ILCs), and dendritic cells—express detectable levels of soluble and membrane-bound TL1A within the lamina propria [25,26].

Functionally, the TL1A–DR3 axis plays a central role in mucosal immunity by coordinating both innate and adaptive immune responses in IBD. TL1A, primarily secreted by antigen-presenting cells stimulated by microbial or cytokine signals, binds to its receptor DR3, which is predominantly expressed on activated CD4+ and CD8+ lymphocytes, ILCs, and regulatory T cells [11]. DR3 contains a death domain in its cytoplasmic region, contributing to inflammatory and apoptotic processes [9,10]. The TL1A-DR3 interaction acts as a co-stimulatory signal that boosts T-cell receptor activation and drives the secretion of key pro-inflammatory mediators, including interferon-γ, interleuchin-17, and TNF-α [26,27,28]. Downstream activation of the nuclear factor kappa-light-chain-enhancer of activated B cells (NF-κB) and mitogen-activated protein kinase (MAPK) pathways further reinforces T helper (Th) 1 and Th17 differentiation, thus sustaining chronic inflammation [29,30,31]. TL1A additionally regulates the balance between regulatory and effector T cells: while low TL1A levels help preserve immunosuppressive Treg function, excessive TL1A production renders effector T cells resistant to Treg-mediated control, shifting immune homeostasis toward a more pro-inflammatory state [32,33].

On the other hand, decoy receptor 3 (DcR3), a soluble member of the TNF receptor superfamily, functions as a decoy receptor by binding TL1A without eliciting downstream signaling. By sequestering TL1A, DcR3 inhibits its interaction with DR3, thereby attenuating TL1A-mediated immune activation and contributing to the modulation of inflammatory responses [20].

The significance of TL1A:DR3 signaling has been underscored by transgenic mouse models that constitutively overexpress Tl1a in either myeloid or lymphoid cells. All such mice develop a pattern of chronic inflammation with patchy distribution in the terminal ileum, with histological features reminiscent of CD.

Other work has demonstrated that *T11a* overexpression exacerbates chronic colitis by perturbing epithelial barrier function through the disruption of tight junctions and facilitating luminal bacterial translocation. In a dose-dependent manner, Tl1a can enhance transcellular microbial uptake through PI3K/Akt-dependent pathways, even in the absence of overt epithelial injury [23].

Conversely, it has been shown that Dr3-deficient mice develop more severe inflammation upon challenge with dextran-sodium sulphate (DSS), in comparison to wild-type controls. One proposed explanation involves the expression of DR3 by intestinal epithelial cells (IECs), which has been demonstrated in both mice and humans. In the absence of DR3, IECs undergo atypical proliferation and modifications of tight junction proteins, including Claudin-1 and zonula occludens-1, resulting in elevated intestinal permeability. Upon DSS administration, the tissue repair capacity of DR3-deficient animals is profoundly impaired, and bacterial translocation is substantially increased, thereby worsening colonic inflammation [34].

Additional work has highlighted the capacity of TL1A to support antimicrobial defenses at the intestinal surface. Recent findings show that TL1A:DR3 engagement is crucial for optimal bacterial uptake and intracellular clearance in human macrophages, a process reliant on autocrine/paracrine loops and influenced by *TNFSF15* variants [35]. Collectively, these studies confirm the integral function of TL1A:DR3 signaling in both immunity and barrier integrity, shedding light on how disruptions within this axis may give rise to sustained inflammation in IBD.

While blockade of the TL1A–DR3 pathway holds therapeutic potential for T cell-mediated autoimmune disorders and inflammation, evidence from DR3-deficient mice highlights possible immunological risks. This signaling axis plays a non-redundant role in costimulating T cell proliferation and cytokine production, particularly in response to dendritic cell-derived TL1A. Notably, Dr3-deficient mice exhibited reduced accumulation and cytokine production of effector T cells in target organs and were protected from immunopathology in models such as experimental autoimmune encephalomyelitis and allergic lung inflammation [10]. This suggests that while TL1A-DR3 blockade may limit tissue-damaging immune responses, it could also impair local T cell-mediated immunity needed for effective responses in inflamed tissues. Therefore, chronic inhibition of TL1A-DR3 signaling might compromise protective immune functions at sites of inflammation, potentially increasing susceptibility to certain infections or affecting tissue immune surveillance.

## 3. TL1A/DR3-Driven Fibrotic Remodeling

Beyond its well-characterized pro-inflammatory role, TL1A has emerged as a major driver of fibrosis in IBD (Figure 1). Fibrosis arises when the normal reparative processes of the intestinal mucosa fail, leading to excessive extracellular matrix deposition in response to persistent inflammatory activity in the bowel wall. Early in the disease, fibrogenesis is principally inflammation-driven, but subsequent self-sustaining loops can perpetuate fibrotic remodeling independently of ongoing inflammation [36].

Evidence of a direct link between TL1A:DR3 signaling and fibrogenesis is provided by the robust expression of both TL1A and DR3 in human subepithelial intestinal myofibroblasts (SEMFs). This expression is further heightened when SEMFs are exposed to supernatants from inflamed epithelial cultures or mucosal samples from patients with CD [19]. In murine models, transgenic overexpression of *Tl1a* consistently leads to hypertrophy of the muscularis propria and localized fibrosis, accompanied by heightened collagen deposition and increased production of Tgfβ1 [37,38].

Notably, TL1A can activate fibroblasts via DR3 signaling in a manner that is largely independent of immune cell-mediated pathways. Upon engaging DR3, TL1A triggers Rho GTPase-dependent mechanisms that drive collagen synthesis, α-smooth muscle actin (α-SMA) expression, and enhanced fibroblast migration. This response is amplified by the upregulation of IL-31 receptor α (IL-31Ra), which further sensitizes fibroblasts to additional profibrotic cytokines. In experimental systems, constitutive overexpression of Tl1a induces spontaneous ileitis alongside marked intestinal fibrosis, characterized by significantly increased collagen deposition [39]. In contrast, anti-TL1A monoclonal antibody therapy effectively reverses such fibrotic changes by inhibiting key fibrotic mediators (including connective tissue growth factor, IL-31Ra, and TGFβ1) [40], underscoring the potential impact of blocking this pathway on fibrotic progression.

Additional mechanistic insights implicate epithelial-mesenchymal transition as another pathway linking chronic inflammation with fibrosis progression: in mice colonic epithelial cells, Tl1a-induced epithelial-mesenchymal transition involves the loss of epithelial markers (notably E-cadherin) and the acquisition of mesenchymal markers such as Ferroptosis suppressor protein (Fsp1) and α-smooth muscle actin [41].

Finally, recent data suggest that TL1A-driven fibrosis also depends on microbial co-stimulation, implicating specific gut bacterial strains in fibroblast activation in susceptible hosts. Germ-free mice engineered to overexpress Tl1a display minimal collagen accumulation unless colonized with specific pathogen-free microbiota, indicating a critical requirement for microbial signals in initiating and sustaining fibrotic remodeling. Streptococcus and Lactobacillus species appear particularly potent inducers of fibroblast activation in vitro, eliciting the upregulation of α-SMA and collagen I [42].

Because TL1A influences both inflammatory and profibrotic processes, efforts to target this axis may have a dual benefit in IBD. Clinical trials evaluating anti-TL1A monoclonal antibodies show not only improved control of inflammation but also reductions in fibrotic biomarkers. Furthermore, interventions aimed at modulating the gut microbiome could augment these antifibrotic effects, providing a comprehensive approach to address the complex pathophysiology of IBD.

## 4. Evidence from Clinical Trials on the Use of Anti-TL1A Drugs in IBD

Many studies have assessed the potential of anti-TL1A drugs for the treatment of IBD (Table 1).

In the first-in-human phase 1 trial [43], PF-06480605, a fully human immunoglobulin (Ig) G1 monoclonal antibody targeting TL1A, was administered to healthy volunteers to assess its safety, tolerability, pharmacokinetics, pharmacodynamics, and immunogenicity. Ninety-two adult participants were enrolled; 60 were assigned to a single ascending dose portion, receiving one intravenous infusion ranging from 1 mg to 800 mg, and 32 were enrolled in a multiple ascending dose phase, where they received either three subcutaneous doses of up to 300 mg or three intravenous doses of 500 mg administered every two weeks. Across all dosing levels, 45 treatment-emergent adverse events were reported in 21 participants who received active drug during the single ascending dose portion, compared with 20 events in 13 individuals who received placebo. In the multiple ascending dose portion, 44 events arose in 17 subjects on active treatment and 17 in 8 subjects on placebo. Despite these observations, no deaths, serious adverse events, or dose reductions occurred, and the agent was deemed well tolerated up to 800 mg intravenously in a single infusion or 500 mg in three consecutive infusions. Clearance appeared faster at lower doses, suggesting potential target-mediated mechanisms at those concentrations, while pharmacokinetic parameters at higher doses were consistent with typical profiles seen for monoclonal antibodies, including a terminal half-life ranging from about 6 to 23 days. A high rate of immunogenicity was noted, with a large fraction of participants—from 50% to 100%, according to the cohorts—developing anti-drug antibodies. However, at doses nearing the upper range, these antibodies did not appear to reduce drug exposure in a clinically meaningful way. Total soluble TL1A concentrations, measured as an indicator of target engagement, were consistently higher in the cohorts given PF-06480605 compared to placebo, indicating effective binding to the target.

Two subsequent phase 2 studies, TUSCANY and ARTEMIS-UC, explored different monoclonal antibodies directed against TL1A in adult patients with IBD refractory or intolerant to standard and/or advanced therapies.

The TUSCANY trial was a phase 2a [44], multicenter, single-arm study that evaluated the safety, tolerability, and preliminary efficacy of PF-06480605 for the treatment of moderate-to-severe UC. The study involved 50 participants who received 500 mg intravenous PF-06480605 every two weeks for a total of seven doses, with a three-month follow-up period. The primary safety endpoint was the incidence of adverse events, while the primary efficacy endpoint was endoscopic improvement at week 14, defined by a Mayo endoscopic subscore of 0 or 1 without friability. Secondary endpoints included endoscopic remission, clinical remission, and other measures such as changes in partial Mayo score over time. Histologic endpoints were evaluated on colonic biopsy samples at baseline and again at week 14, focusing on the proportion of patients achieving minimal histologic disease. Out of 50 participants, 42 completed the study. Participants were predominantly male (56%), with a mean age of 40 years. Most had prior exposure to corticosteroids (92%) and TNF-α inhibitors (72%). Safety assessments revealed 66% of participants (*n* = 33) experienced at least one treatment-emergent adverse event (TEAE), while the total number of TEAEs reported was 109, 18 of which were treatment-related, experienced by 8 (16.0%) patients. Serious adverse events (SAEs) occurred in 6% of participants (*n* = 3), with a total of four SAEs reported. The most common TEAEs were ulcerative colitis exacerbation and arthralgia, each occurring in 12% of participants (*n* = 6). Less common TEAEs included abdominal pain, nausea, nasopharyngitis, pharyngitis, back pain, and alopecia areata, each occurring in 6% of participants (*n* = 3). No deaths or malignancies were reported. Efficacy analysis showed an estimated rate of endoscopic improvement of 38.2% (95% confidence interval [CI] 23.8–53.78%) at week 14, which was statistically significant for the rejection of the null hypothesis, based on the uniformly minimum-variance unbiased estimator method (*p* < 0.001). Remission (total Mayo score ≤ 2 with no individual subscore > 1) and endoscopic remission rates were 24% and 10%, respectively. Minimal histologic disease, defined as a Robarts Histopathology Index ≤ 3.2 or a Geboes Index ≤ 5, was observed in 33.3% and 47.6% of participants, respectively. Pharmacokinetic analysis demonstrated typical profiles for a monoclonal antibody, with sustained target engagement indicated by increased total soluble TL1A levels over time—as PF-06480605, when binding and neutralizing soluble TL1A, is proposed to also stabilize it and also reduce its elimination. Immunogenicity assessments revealed that 82% of participants developed anti-drug antibodies, while 10% developed neutralizing antibodies. However, there were no statistically significant effects of antibody status on efficacy or pharmacokinetics.

ARTEMIS-UC [45], a subsequent phase 2 study, had a more rigorous design for the assessment of efficacy and safety of TL1A inhibition. This multicenter, randomized, double-blind, placebo-controlled trial evaluated the efficacy and safety of tulisokibart, a humanized IgG1 kappa monoclonal antibody binding to TL1A, in patients with moderate-to-severe UC. A total of 178 participants were enrolled and assigned in a 1:1 ratio to receive intravenous tulisokibart (1000 mg on day 1, followed by 500 mg at weeks 2, 6, and 10) or placebo, with stratification based on previous exposure to advanced therapy and a genetic-based diagnostic test to predict likelihood of response to anti-TL1A therapy. In particular, the trial included two cohorts: Cohort 1, which enrolled patients regardless of their status on the genetic test, and Cohort 2, which included only patients with a positive test result. The primary efficacy endpoint was clinical remission at week 12 in Cohort 1, defined as a modified Mayo endoscopic subscore of 0 or 1, a rectal-bleeding subscore of 0, and a stool-frequency subscore of 0 or 1. In Cohort 1, 135 patients were randomized (68 to tulisokibart, 67 to placebo), of whom 128 completed the 12-week induction period. Baseline characteristics indicated a treatment-refractory population, with nearly half having previously received biologic or small-molecule therapies. At week 12, 26% of those receiving tulisokibart achieved the primary endpoint of clinical remission, compared with 1% in the placebo group (*p* < 0.001). Tulisokibart also showed consistent efficacy across the prespecified secondary endpoints: higher rates of endoscopic improvement (37% vs. 6%, *p* < 0.001), clinical response (66% vs. 22%, *p* < 0.001), histologic improvement (46% vs. 28%, *p* < 0.001), and combined histologic–endoscopic mucosal improvement (31% vs. 4%, *p* < 0.001). Continuous disease-activity measures—such as the partial Mayo score and its subscores—favored tulisokibart beginning as early as week 2 and extending through week 12. A prespecified subanalysis examined patients who tested positive on the diagnostic assay, pooling data from both cohorts. Among these 75 patients, a higher proportion of those receiving tulisokibart achieved clinical remission at week 12 compared to placebo (32% vs. 11%). Safety results showed comparable rates of adverse events in the tulisokibart and placebo arms (46% vs. 43%, respectively), and serious adverse events were infrequent. Infections—classified as events of special interest—occurred at similar rates in both groups (18%), and no acute infusion reactions were reported. A post-hoc analysis specifically evaluated the effects of re-induction treatment in patients who did not achieve a clinical response during the initial 12-week induction period [46]. Non-responders from both the tulisokibart and placebo groups entered a new 12-week open-label re-induction phase, receiving the same tulisokibart induction regimen. At Week 14, 41 placebo-treated and 21 tulisokibart-treated non-responders proceeded to re-induction. By Week 26, 48% and 63% of patients who had received 24 weeks of tulisokibart achieved symptomatic improvement and symptomatic response, respectively, while among those who had received 12 weeks of placebo followed by 12 weeks of tulisokibart, 63% and 76% achieved symptomatic improvement and symptomatic response, respectively. Furthermore, no serious adverse events nor new safety signals were identified in patients receiving re-induction.

More recently, preliminary data from subsequent trials on anti-TL1A drugs have been presented.

The phase IIb TUSCANY-2 trial is a multicenter, randomized, double-blind, dose-ranging study enrolling adult patients with moderate-to-severe active UC [47,48]. Patients were randomized to receive monthly subcutaneous injections of PF-06480605 (also known as RO7790121), an anti-TL1A antibody, at doses of 50 mg, 150 mg, or 450 mg, or matched placebo during a 12-week induction period, followed by a 40-week treat-through maintenance phase. A total of 245 patients received at least one dose of study medication, with 228 completing induction, 224 entering maintenance, and 178 completing the entire maintenance period. The trial evaluated both symptomatic improvements and objective histologic-endoscopic endpoints. Early symptomatic relief was observed with PF-06480605 treatment, with improvements in rectal bleeding evident as early as Week 2, when 34% of PF-06480605-treated patients reported no rectal bleeding compared to 20% of placebo-treated patients. Stool frequency improvements progressively increased throughout the induction period, with approximately 60% of PF-06480605-treated patients achieving a stool frequency subscore of 0 or 1 by Weeks 12 and 14, compared to approximately 40% for placebo. Symptomatic remission rates were consistently higher with PF-06480605 versus placebo, beginning at Week 2 (17.5% vs. 8.9%) and increasing to 52.0% versus 31.1% by Week 14, representing a treatment difference of 20.9% (90% CI: 7.4–32.6%). The objective histologic endpoints further supported the clinical benefits of PF-06480605. At Week 14, histologic improvement (Geboes score ≤ 3.1) was achieved in 40.0%, 39.6%, and 32.9% of patients receiving PF-06480605 50 mg, 150 mg, and 450 mg, respectively, compared to just 12.2% with placebo. Similarly, histologic-endoscopic mucosal improvement (Geboes score ≤ 3.1 and endoscopic subscore ≤ 1) was observed in 30.0%, 32.1%, and 25.6% of patients in the respective PF-06480605 treatment groups versus 4.9% with placebo. Notably, these improvements were sustained through Week 56 of the study. Higher rates of histologic and endoscopic remission were also observed with all PF-06480605 doses compared to placebo, with benefits maintained through Week 56. Interestingly, there was no apparent dose-response relationship across the various endpoints. The safety profile was generally favorable, with treatment-emergent adverse events reported in 117 of 245 patients during induction. Serious treatment-emergent adverse events occurred in ten patients during induction, with only two considered treatment-related. Six patients discontinued treatment due to adverse events. PF-06480605 is currently being further evaluated in phase III trials (NCT06589986 and NCT06588855).

The RELIEVE UCCD trial (NCT05499130), a phase 2b randomized, double-blind, placebo-controlled, dose-ranging basket study, assessed the efficacy, safety, and tolerability of duvakitug (TEV-48574), a human IgG1 monoclonal antibody inhibiting TL1A, as an induction therapy in adults with moderate-to-severe UC and CD [49,50]. In the UC cohort, 137 patients with prior inadequate response, loss of response, or intolerance to conventional or advanced therapies were randomized to receive an initial 2250 mg subcutaneous loading dose of duvakitug or placebo, followed by either 450 mg or 900 mg of duvakitug, or placebo, every two weeks. At week 14, clinical remission, assessed via a modified Mayo score, was achieved in 36% and 48% of patients receiving duvakitug 450 mg and 900 mg, respectively, compared to 20% in the placebo group, with placebo-adjusted remission rates of 16% and 27%. Bayesian analysis confirmed a >90% posterior probability that duvakitug was superior to placebo, with a consistent treatment effect in both advanced therapy-experienced and naïve patients. The incidence of adverse events was lower for duvakitug 450 mg (49%) and 900 mg (43%) compared to placebo (52%), with discontinuation due to adverse events occurring in 0% of the 450 mg group, 2% of the 900 mg group, and 5% of the placebo group. In the CD cohort, 138 patients were similarly randomized and treated, with the primary endpoint being endoscopic response, defined as a ≥50% reduction in the SES-CD at week 14. Endoscopic response rates were 26% and 48% for duvakitug 450 mg and 900 mg, respectively, compared to 13% in the placebo group, with placebo-adjusted response rates of 13% and 35%. Bayesian analysis confirmed statistical significance, and efficacy was observed across both advanced therapy-experienced and naïve patients, with a 90% posterior probability. The safety profile was comparable to placebo, with adverse event incidences of 43% for duvakitug 900 mg, 67% for 450 mg, and 48% for placebo, and discontinuation rates of 2% for duvakitug 900 mg and placebo, and 9% for duvakitug 450 mg. These findings represent the first randomized, placebo-controlled induction study of an anti-TL1A monoclonal antibody in CD.

Taken together, the results support the hypothesis that inhibiting TL1A might provide meaningful relief for individuals who have not improved on standard treatment options.

## 5. TL1A as a Potential Target in the Gut–Synovium–Skin Axis

TL1A upregulation is not restricted to the intestine, but extends to affected skin and joints as well, placing this cytokine at the nexus of a gut–synovium–skin axis. This broad pathogenic role underscores TL1A’s potential as a therapeutic target in individuals with IBD who develop extraintestinal manifestations or other immune-mediated inflammatory diseases (IMIDs), including rheumatoid arthritis (RA), ankylosing spondylitis (AS), psoriasis, and psoriatic arthritis.

Multiple lines of evidence highlight TL1A’s importance in RA. Elevated concentrations have been documented in synovial fluid, serum, and inflamed joint tissue, correlating with the presence of autoantibodies (i.e., rheumatoid factor and anti-cyclic citrullinated peptide) and driving Th1/Th17 pathways by enhancing IL-17, IL-6 and IFN-γ production [51,52,53]. In collagen-induced arthritis models, Tl1a blockade reduces disease severity by up to 60%, abrogating synovial inflammation and bone erosions [54]. Clinical observations reveal that anti-TNF therapies often lower TL1A levels, suggesting the involvement of TNF-α in the TL1A pathway [55]. Furthermore, independent investigations repeatedly confirm that, in patients with RA, TL1A and its decoy receptor DcR3 are elevated in both peripheral blood and inflamed joint compartments [52,56]. In RA, nuclear TL1A staining has been observed in synoviocytes and inflammatory cells, mirroring a phenomenon also documented in psoriatic lesions [57]. Taken together, these findings illustrate a critical local role for TL1A in driving synovial inflammation and tissue damage.

A similar pattern emerges in AS. Konsta et al. demonstrated that soluble TL1A concentrations are significantly higher in anti-TNF-α naïve patients with AS, compared to healthy controls, whereas those already receiving anti-TNF-α therapy show nearly normalized TL1A levels. Higher TL1A levels have been linked to higher disease activity scores, substantiating TL1A as both a potential biomarker and a driver of pathogenic processes in axial spondyloarthropathy [55].

Psoriasis and psoriatic arthritis likewise exhibit marked involvement of TL1A. Both diseases are frequently associated with IBD [58], and studies point to a significant upregulation of the TL1A/DR3/DcR3 system in the pathogenic cell populations of active psoriatic lesions [59]. Nuclear localization of TL1A appears in chronic inflammatory states such as psoriasis, yet is absent in healthy skin, raising the hypothesis that persistent inflammatory stimuli induce or sustain this atypical intracellular localization [57]. In functional terms, TL1A synergizes with IL-23 to amplify IL-17 production, promoting epidermal hyperproliferation. Treatment effectiveness, in psoriasis, often correlates with a reduction of circulating TL1A [60], further implicating this pathway in cutaneous immunopathology.

Beyond the skin, joints, and bowel, the TL1A/DR3 axis influences other immune-mediated conditions. Notably, experimental autoimmune uveitis models exhibit elevated Dr3 and Tl1a during early, active disease, with both returning toward baseline as inflammation resolves. Recombinant TL1A added to CD4⁺ T cells increased IL-17 secretion, suggesting a link between DR3/TL1A and the IL-17-dependent arm of ocular autoimmunity [61].

A critical aspect of TL1A biology is that its signaling can be highly responsive to low-grade stimulation. This responsiveness makes it an attractive therapeutic target for controlling lingering, smoldering inflammation—particularly in patients managing multiple comorbid IMIDs or extraintestinal manifestations in the setting of IBD [62]. While more potent immunosuppressants may be employed initially to quell severe disease flares, adjunctive anti-TL1A therapy may help maintain long-term disease control by curbing persistent low-level inflammation. This rationale fits within the broader concept of combination or dual-targeted therapies, which aim to achieve robust and sustained remission in complex immunological diseases.

## 6. TL1A as a Mediator in Other Diseases

TL1A has emerged as a versatile mediator implicated in several conditions characterized by persistent low-grade inflammation, underscoring its potential impact beyond classical immune-mediated disorders.

In colorectal cancer (CRC), TL1A fosters metastatic progression by facilitating epithelial-to-mesenchymal transition, which enhances the migratory and invasive capabilities of cancer cells, through the modulation of the TGF-β/Smad3 signaling. In vitro experiments demonstrated that TL1A knockdown suppressed CRC cell proliferation and metastatic potential, accompanied by altered expression of epithelial-to-mesenchymal transition biomarkers such as E-cadherin and vimentin [63]. This evidence may position TL1A as a possible therapeutic target for halting cancer dissemination.

In obesity, TL1A cooperates in a paracrine loop that disrupts adipose tissue homeostasis, impairing insulin signaling in adipocytes and promoting lipid-laden macrophages, thereby exacerbating metabolic complications [64]. Within the diabetic retinopathy milieu, TL1A expression is initially reduced but surges in advanced stages, possibly mitigating neovascularization by countering the proangiogenic drive of vascular endothelial growth factor (VEGF), even though its protective role may be overshadowed by persistent high VEGF level [65,66].

Furthermore, dysregulated TL1A activity has been implicated in the progression of atherosclerosis [67]: in patients with RA higher TL1A is observed to correlate strongly with increased carotid atheromatous plaque progression. Conversely, individuals exhibiting low TL1A and undetectable DcR3 develop significantly fewer new carotid plaques over a 3.5-year period. Intriguingly, however, studies in apoE-deficient mice reveal that Tl1a can inhibit lesion formation by modulating vascular smooth muscle cell behavior, suggesting a context-dependent influence of TL1A on vascular pathology [68].

In eosinophilic asthma, TL1A heightens airway inflammation through group 2 innate lymphoid cells (ILC2s) and drives structural changes in the airways by inducing fibrotic pathways in stromal cells, thereby contributing to steroid-resistant disease phenotypes [69,70].

Collectively, these findings underscore the multifaceted actions of TL1A across a range of chronic pathologies and support its potential status as a pivotal therapeutic target for conditions extending beyond the realm of traditional immune-mediated diseases.

## 7. Conclusions

TL1A stands out as a pivotal cytokine at the intersection of chronic inflammation and fibrotic remodeling across multiple disease states. Its ability to potentiate both innate and adaptive immune responses, promote fibroblast activation, and modulate barrier integrity underscores its far-reaching pathogenic capacity in IBD, where it fuels persistent intestinal inflammation and drives fibrostenotic complications. Beyond the gut, TL1A’s expression and function in rheumatological, dermatological, and other immune-mediated pathologies attest to its broad significance. Recent clinical trials in UC have demonstrated encouraging efficacy and safety profiles for anti-TL1A agents, highlighting both inflammation control and potential antifibrotic benefits. These findings strengthen the rationale for pursuing TL1A blockade as a precision-medicine strategy, particularly in treatment-refractory patients or those with overlapping comorbidities. Looking ahead, refining patient selection through companion diagnostics and exploring combination approaches that target TL1A alongside other key pathways may further enhance outcomes, positioning TL1A inhibition as a promising advance in the evolving landscape of inflammatory and fibrotic disease management.

## Figures and Tables

**Table 1:** Completed or actively recruiting clinical trials of anti-TL1A drugs in IBD. i.v., intravenously; s.c., subcutaneously; Q4W, every four weeks; Q2W, every two weeks; UC, ulcerative colitis; CD, Crohn’s disease; SAD, single ascending dose; MAD, multiple ascending dose.

Trial ID/Name	Design	Intervention	Comparator	Patient Cohort	Primary Outcome	Status
NCT01989143	Phase 1	PF-06480605 1, 3, 10, 30, 100, 300, 600, or 800 mg i.v., or MAD, PF-06480605 3 × 500 mg i.v., or 3 × 30 mg, 3 × 100 mg, or 3 × 300 mg s.c. Q2W for 3 doses	Placebo	Healthy subject	Safety, tolerability, pharmacokinetics, pharmacodynamics, and immunogenicity of SAD and MAD	Completed
NCT02840721 TUSCANY	Phase 2a, single-arm study	PF-06480605 500 mg i.v. Q2W, 7 doses total	/	Moderate-to-severe UC	Safety, tolerability, and preliminary efficacy (endoscopic improvement at week 14)	Completed
NCT04996797 Artemis-UC	Phase 2a	Tulisokibart 1000 mg i.v. on day 1, followed by 500 mg i.v. at weeks 2, 6, and 10	Placebo	Moderate-to-severe UC	Clinical remission at week 12	Active, not recruiting
NCT04090411 TUSCANY-2	Phase 2b	PF-06480605 50 mg s.c. Q4W or 100 mg s.c. Q4W or 150 mg s.c. Q4W	Placebo	Moderate-to-severe UC	Safety, tolerability, and clinical remission at week 14	Completed
NCT05499130 RELIEVE UCCD	Phase 2b	Initial 2250 mg s.c. loading dose of duvakitug (TEV-48574), followed by 450 mg s.c. Q2W or 900 mg s.c. Q2W	Placebo	Moderate-to-severe UC and CD	Safety, tolerability, clinical remission (UC) and endoscopic response (CD) at week 14	Completed
NCT05668013 RELIEVE UCCD LTE	Phase 2b LTE	Drug: TEV-48574 dose regimen A Drug: TEV-48574 dose regimen B	/	Moderate-to-severe UC and CD	Clinical remission (UC) and endoscopic response (CD) at week 24	Active, not recruiting
